# Gatekeeper™ Prostheses Implants in the Anal Canal for Gas Incontinence and Soiling: Long-Term Follow-Up

**DOI:** 10.3390/jcm13206156

**Published:** 2024-10-16

**Authors:** Jaume Tur-Martinez, Laura Lagares-Tena, Juan Hinojosa-Fano, Antonio Arroyo, Albert Navarro-Luna, Arantxa Muñoz-Duyos

**Affiliations:** 1 Department of General Surgery, Hospital Universitari MútuaTerrassa, Universitat de Barcelona, 08221 Terrassa, Spain; 2 Colorectal Surgery Unit, Department of General Surgery, Hospital Universitario de Elche, Universidad Miguel Hernández, 03202 Elche, Spain

**Keywords:** Gatekeeper™ prostheses, bulking agents, faecal incontinence, gas incontinence, soiling, outcome

## Abstract

**Introduction**: Although several treatments for faecal incontinence are available, gas incontinence (GI) and soiling are difficult to manage. The aim of this study is to evaluate Gatekeeper™ for this subtype of faecal incontinence. **Methods**: Prospective single-centre case series. Patients with mainly soiling and/or GI were treated with polyacrylonitrile prostheses. An evaluation was performed with a 3-week continence diary. St. Mark’s score and a Visual Analogue Scale (VAS) were used to study the patient’s continence perception and surgical satisfaction, at baseline and 1, 3, 6, 12, and 24 months postoperatively. 3D-Endoanal Ultrasound and Anorectal Manometry were performed at baseline and postoperatively. **Results**: A total of 13 patients were enrolled (11 women), aged (median (IQR)) 62 (13) years, and all implants were uneventful. A significant reduction in soiling and GI episodes was documented at 1 year, 7 (18) baseline days of soiling/3 weeks vs. 2 (4) (*p* = 0.002); 13 (13) baseline episodes of GI/3 weeks vs. 4 (10) (*p* = 0.01). This improvement was correlated with a significant increase in VAS (0–10), 3 (2, 5) baseline vs. 7 (1, 5) (*p* = 0.03), and maintained throughout the follow-up. There was complete remission or significant improvement defined as >70% reduction in gas and soiling days in 6 patients at 2 years follow-up. Soiling episodes were reduced ≥70% in 8/11 patients (72.7%). Nine (70%) patients would repeat the treatment. **Conclusions**: Gatekeeper™ is a safe, minimally invasive treatment for soiling and GI. A significant reduction in soiling and GI was observed in our series, with a better response to soiling. Most of the patients would repeat the treatment. Other studies are needed to confirm these findings in this subgroup of FI patients.

## 1. Introduction

Faecal incontinence (FI), affecting between 2% and 10.8% of the general population [1,2], has a multifactorial etiology with a wide range of symptoms. Gas incontinence (GI) and soiling defined as the involuntary leakage in small amounts that occurs without the patient being aware of or able to control it, are often considered as mild FI. However, they can significantly impact patients’ quality of life. Recently, European guidelines for the management of FI [3] have emphasized the importance of individualised treatment strategies based on specific patient pathophysiology and symptoms. With the numerous treatment options now available for FI, it may be time to consider categorising the syndrome into distinct clinical presentations and testing different therapeutic approaches to improve outcomes.

Several treatment options exist, ranging from conservative to surgical approaches, which can be tailored according to the severity of symptoms. Non-surgical treatments include dietary modifications, pelvic floor exercises, and biofeedback therapy. Transanal irrigation (TAI) has also shown promise in managing FI, with a recent observational study reporting positive outcomes for FI patients using TAI [4]. These therapies, along with sphincteroplasty and sacral nerve stimulation (SNS), offer a wide array of treatment choices for different patient needs.

Among minimally invasive methods, injectable bulking agents have been extensively used [5,6,7,8,9,10,11,12,13], though the varied materials injected in small case series have produced mixed results [7,14,15,16].

In 2011, Ratto et al. introduced Gatekeeper™, a self-expandable polyacrylonitrile prosthesis implanted into the upper-middle intersphincteric space of the anal canal, showing promising results [17]. The prostheses absorb fluid after implantation, expanding to create bulk and support, which improves sphincter closure and control. Complications can include infection, pain, migration of the prostheses, or failure to improve symptoms.

This study aims to present the clinical outcomes and long-term follow-up of Gatekeeper™ treatment in a homogeneous group of patients characterised by GI and/or soiling as their primary, bothersome symptom.

## 2. Method

This is a prospective, single-centre case series, carried out in a university hospital from February 2014 to March 2018, and reported according to the STROBE statement for cohort studies [18]. All patients referred to the centre who met the inclusion criteria were followed up using a specifically designed protocol. The study protocol was approved by the Ethics Committee. Patients selected for the study were informed about the study and signed the informed consent.

Patients with previous history of chronic diarrhoea were evaluated by the gastroenterologists and if needed, fibre supplementation or other medical treatment was used to obtain a Bristol score of 3–4 before entering the study.

Inclusion criteria: patients older than 18 years old, with at least 6 months of GI and/or soiling, non-responding to conservative treatment and biofeedback.

Exclusion criteria: patients with complete solid and liquid FI, common Bristol score > 4, malignant diseases, chronic diarrhoea, inflammatory bowel disease, acute anorectal disease, rectal prolapse, neurological disease, obstructive defecation syndrome, low anterior resection syndrome, previous pelvic radiation, any anaesthetic contraindication, St. Mark’s score > 12 or external anal sphincter lesion > 30°.

The procedure was performed under general anaesthesia with a laryngeal mask, by a senior colorectal surgeon with more than 10 years of experience in perianal procedures. Patients were prepared with 1 to 2 enemas and antibiotic prophylaxis was administered to all patients with 4 g/0.5 g of Piperacillin/Tazobactam. Six polyacrylonitrile prostheses (THD Gatekeeper™ Delivery System, THD SpA, Correggio, Italy) were implanted under 3D-EUS control through a 5 mm perianal incision at 1, 3, 5, 7, 9, and 11 o’clock in the lithotomy position, and located in the upper-middle intersphincteric space of the anal canal, using the system described by Ratto et al. [19].

Patients were discharged on the same day and recommended to avoid any trauma or sexual practice during the first week after implantation. No postoperative antibiotics were prescribed. All patients were followed up 10 days after surgery, at 1, 3, 6, and 12 months, and then annually.

The follow-up was conducted using three primary tools:

1. A three-week continence diary, specifically recording GI and soiling episodes.
2. The St. Mark’s Incontinence Score (0–24) [20], which evaluates the severity of FI through several variables: the frequency of solid and liquid stool leakage, urgency, control of flatus, usage of protective pads, usage of astringent medications, and the impact on quality of life. Higher scores reflect more severe incontinence.
3. A Visual Analogue Scale (VAS), where patients rated their continence from 0 (awful) to 10 (perfect), to capture their subjective perception of the condition.

Moreover, at the end of the follow-up, a survey was conducted asking patients about their satisfaction with the procedure using another Visual Analogue Scale (VAS) being 0 very dissatisfied and 10 very satisfied, and their willingness to repeat the treatment.

Moreover, patients were classified into two clinical response categories at the last follow-up: 1. Improvement of GI > 70% (yes/no). 2. Improvement of SI > 70% (yes/no).

At baseline and 3 months postoperative anorectal manometry, testing of pressures and volumes was performed using a four-channel manometer. Additionally, 3D-EAUS was performed preoperatively, and one month after surgery, to check the prostheses’ locations. Prostheses migration was defined as displacement of the prosthesis to the suprasphincteric space from the medium anal canal, detected during the follow-up.

Data analysis was carried out using the statistical package SPSS version 20. Results are presented using the median and interquartile range (VAS, St. Mark’s score, and the number of FI episodes). Statistical analysis was conducted using the Wilcoxon test. *p* values represent the statistical difference between data with a value of <0.05 considered to show statistical significance.

## 3. Results

Out of 197 visited patients with FI, 15 were eligible to be treated with Gatekeeper™. Eleven women and two men with a median age of 62 (13) years accepted the treatment. The other two patients preferred to continue with just conservative treatment. Nine patients had previously undergone anal surgery (haemorrhoidectomy, fistulectomy, and internal lateral sphincterotomy) or gynaecological surgery (transvaginal hysterectomy and caesarean section) (Table 1). Eight women had obstetric backgrounds (instrumental vaginal deliveries), with a median of 2 (1, 5) vaginal deliveries.

All patients were previously treated with biofeedback without success. Only one patient required previous treatment with fibre supplementation because of diarrhoea. One patient with more severe FI at the beginning of her treatment history was also treated with sphincteroplasty and subsequent sacral neuromodulation, with unsolved GI and soiling at her inclusion in the study (Table 1).

Patients had been suffering from GI or soiling for a median of almost 3 years (36 (42) months before receiving treatment. Baseline clinical features are shown in Table 2. Surgery time was 38 (42) minutes with no intraoperative incidents.

All the Gatekeeper™ procedures were uneventful, with no adverse effects or complications recorded. No cases of prostheses or wound infection occurred.

The evolution of the clinical parameters throughout the follow-up is shown in Table 3. A significant reduction in soiling episodes was documented from the first-month follow-up and, at 1 year, it was reduced to 2 (4) episodes for 3 weeks from 7 (18) at baseline (*p* = 0.002). GI was also significantly reduced from 13 (13) episodes of GI for 3 weeks at baseline, to 4 (10) at 1-year follow-up (*p* = 0.01). This improvement was correlated with a significant increase in VAS from 3 (2, 5) at baseline to 7 (1, 5), *p* = 0.03. This tendency was maintained throughout the entire follow-up.

An improvement in the St. Mark’s score was seen in the first month of follow-up and maintained during the third and sixth months of follow-up, but slight progressive worsening in the St. Mark’s score posteriorly occurred, with 2 years follow-up of the St. Mark’s score close to the baseline. However, patients remained clinically better than at baseline, with a statistical significance in VAS results.

The evolution of the patients from baseline to the last follow-up is shown in Table 4. At the last follow-up, >70% reduction in GI was achieved in six patients with significantly better results in the VAS and St. Mark’s score. Moreover, the soiling episodes were reduced by ≥70% in 8 out of 11 patients (Table 4).

Regarding patient satisfaction, nine of the patients would repeat the treatment, and only four patients were disappointed.

Baseline manometry values showed low resting pressures (<50 mmHg in women; <80 mmHg in men) in all 13 patients and low squeeze pressure (defined as less than double resting pressure) in 3 patients (Table 5). No significant changes were observed in manometry resting pressure and median squeeze pressure. Regarding the rectal sensitive values, a significant increase in the first sensation volume was detected during follow-up (Table 5).

**Figure 1:**
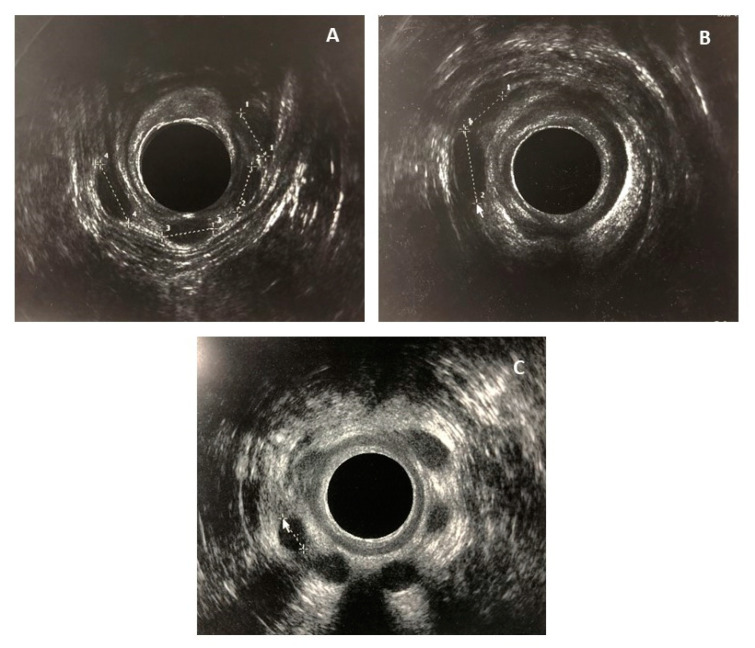
Prostheses location in two different patients. Pictures (**A**,**B**) belong to the same patient with 4 migrated prostheses: (**A**) four prostheses in the suprasphincteric space; (**B**) two prostheses in the medium anal canal. Picture (**C**) shows a patient with six prostheses in the medium anal canal.

During the follow-up, migration of one or more prostheses to the suprasphincteric space was observed in nine patients (Figure 1), with a median of two (2) prostheses migrated per patient. No prostheses migrations towards the anal verge were registered.

## 4. Discussion

This study has focused on testing the efficacy of Gatekeeper™ in a subgroup of patients with only GI and/or soiling. Soiling episodes were reduced by ≥70% in 72.7% of patients with previous symptoms, and GI was reduced by ≥70% in almost half of the patients at two years follow-up, with a good correlation with the improvement of patients’ subjective perception of continence in our series. Ratto et al. analysed the effect of Gatekeeper™ in treating patients with more severe and wider spectrum FI symptoms, reporting an improvement of over 75% for soiling and GI at 1-year follow-up [17]. In this way, Jabbar et al. [21] have published a sustained clinical improvement beyond 5 years for a subgroup of patients with passive FI symptoms, but no previous series have been focused on patients presenting with just GI or soiling. With FI being such a wide-spectrum syndrome, it is possible that a time may come to consider subdividing it into different clinical presentations. Other authors have evaluated the progression of gas incontinence in patients with broader symptoms, such as mixed types of faecal incontinence [22], but no study has focused on patients with this specific presentation of only gas incontinence and/or soiling.

Based on patients’ diaries, which can be considered the most objective tool to evaluate FI, an overall consistent significant decrease in soiling and GI was observed in our series throughout the follow-ups. Moreover, to better analyse our patients’ clinical situations and identify how many patients gained real benefit from the treatment, patients were classified into four groups depending on the percentage of reduction of their symptoms. Traditionally, the most widely used cut-off point to determine improvement in FI has been a 50% reduction in the number of FI episodes [22,23]. However, in our opinion, reducing the number of episodes of soiling or GI to 50% may not imply real changes in quality of life as symptoms are milder but persist. The improvement cut-off was consequently increased to 70%, as already suggested in previous multicentre studies [19,23]. With this ambitious cut-off point, at 1-year follow-up, 53.8% of our series had at least a 70% improvement in their soiling and GI episodes, with 46% of patients in this category being at two-year follow-up. These results are similar to those reported by Ratto et al. for patients with all types of FI combined [19].

The multifactorial nature of FI makes it challenging to find an accurate treatment for each patient. The symptoms are wide-ranging, from soiling or urge to gas, and liquid or solid incontinence [24]. Although the most severe grade of FI is solid incontinence, soiling and GI can be very stressful and invalidating, and these symptoms are usually underestimated in international scores, as happens with soiling in St. Mark’s score [20].

In a more recent paper including a total of 49 patients with FI [22], wider criteria were used, including patients with low anterior resection syndrome, idiopathic FI, and secondary to sphincter lesions, but good results were obtained with a significant improvement in the St. Mark’s score (greater than 50% from baseline values) in 48% of patients. This tendency was maintained after 2.7 years of follow-up. In contrast, the present study notes a lack of significant improvement, likely because GI contributes only 4 points on the 24-point scale, and soiling is not included. This emphasizes the need for multimodal evaluation, including continence diaries, to better capture changes.

No manometric pressure changes were found in our series. A recent study showed an increase in the external anal sphincter contractility after the Gatekeeper™ implant, which was correlated with the ability to defer defecation for more than 5 min in a high proportion of patients [25]. These results were not reproduced in our series. The mild but significant increased volume in the first rectal sensation in terms of rectal sensitivity could help patients in deferring defecation, but the clinical significance of this finding is uncertain.

Unanswered questions include the implication of the prosthesis’s migration on the patient’s evolution, and future research could be focused on the etiopathogenity of these migrations and how to avoid them.

A prosthesis displacement outside the intersphincteric space was seen in 69.2% of our patients. We were not able to investigate the association between the displacement and the efficacy drop in our series. This phenomenon has previously been documented by another group, with an index of prostheses displacement of around 70% [26], in line with our results. Other authors have also reported a migration of the prostheses in 52% of patients, which seems to be directly related to bad functional results [22]. This is one of the reasons why this procedure must always be performed under EUS control to ensure that the prostheses are implanted in the intersphincteric space. Moreover, small body movements in the first postoperative hours may cause prostheses dislocation. For this reason, a change in our clinical practice was introduced and presently, all patients remain hospitalised for 24 h postoperatively.

The implantation of polyacrylonitrile prostheses into the upper-middle intersphincteric space to treat patients with soiling and GI yields good results and is, in our experience, an easy and safe technique, as previously reported [17,19,22,27,28,29].

The use of a VAS to measure patients’ subjective perception of continence is key in evaluating such a complex disorder, as other authors have already suggested previously [24]. This parameter has shown consistent and well-correlated results with a reduction of GI and soiling episodes. Moreover, a final question about patients’ satisfaction with the treatment was introduced in this study, and most of the patients (70%) would be willing to repeat the treatment, although, in some, the improvement was not considered significant based on objective variables. In our opinion, this may be explained by the summative effect of the following factors as a part of the clinical improvement: a minimally invasive technique, with very low morbidity and quick recovery, considered positive by the patients.

The main limitations of this study are the small sample size and the lack of a quality-of-life evaluation using standardised international scoring systems [30,31,32]. However, this limitation was partially addressed by the use of a VAS assessment, which provided a subjective measure of patients’ perceived continence. While VAS offers valuable insight, future studies would benefit from integrating validated, internationally recognised quality-of-life scores to enhance the robustness of the evaluation.

Moreover, this is the first study evaluating this subgroup of FI patients, and patients were assessed with different tools, conferring more strength to the results obtained.

An evolution of this therapeutical option is already available. Sphinkeeper™ consists of implanting 10 polyacrylonitrile prostheses that are a bit longer and thicker. These treatment results have already been published in some series [33,34], and they may also be useful in this specific indication, i.e., GI and soiling. Further studies will be necessary to test this.

## 5. Conclusions

This study presents new evidence on the effectiveness of Gatekeeper™ in a subgroup of patients classified as having mild FI, particularly those with GI and soiling, conditions that have been scarcely studied. The effectiveness was assessed using multiple tools, adding consistency to the findings. Gatekeeper™ is a safe, easy, minimally invasive, and reproducible treatment for soiling and GI. Comprehensive preoperative and follow-up evaluations are recommended. Further long-term prospective studies are necessary to confirm these findings in this specific FI subgroup.

## Figures and Tables

**Table 1:** Patients’ backgrounds.

	*n*
Age	62 (13)
Sex (women:men)	11:2
Previous anal surgery	6 (46%)
Gynaecological surgery	5 (38%)
Instrumented vaginal deliveries	8 (61%)
Other risk factors (Diabetes mellitus, Right colonic resection)	2 (15%)
Urinary incontinence	4 (30%)
**Previous treatments:**	
Anal Sphinteroplasty + SNM ^†^	1 (8%)
Biofeedback	13 (100%)
Fiber supplementation	1 (8%)

^†^ SNM: sacral neuromodulation. Data: Median (IQR), *n* (%).

**Table 2:** Baseline Clinical features.

Clinical Features	*n*
FI ^†^ evolution time (months) Bristol Score	36 (42) 4 (0, 5)
Soiling (days/3 weeks)	7 (18)
Gas Incontinence (days/3 weeks)	13 (13)
Liquid FI (episodes/3 weeks)	0 (0)
Solid FI (episodes/3 weeks)	0 (0)
Urgency (episodes/3 weeks)	0 (4)
Sometimes urgency	5 (38%)
Never urgency	8 (62%)
St. Mark’s score (0–24)	8 (4)
VAS ^‡^ (0–10)	3 (2, 5)
**Need to wear pad**	
Always	5 (38%)
Sometimes	4 (31%)
Never	4 (31%)
**Endoanal US ^§^**	
No lesion	9 (69%)
IAS ^¶^ lesion ^‡‡^	3 (23%)
IAS & EAS ^††^ lesion ^§§^	1 (8%)

^†^ FI: faecal incontinence, ^‡^ VAS: Visual Analogue Scale, ^§^ US: ultrasound, ^¶^ IAS: internal anal sphincter, ^††^ EAS: external anal sphincter, ^‡‡^ Grade of IAS lesions: 1 patient with 90° defect, 1 patient with 40° defect, and 1 patient with 130° defect. ^§§^ Grade of IAS and EAS lesion: 20° EAS defect and IAS damaged in several locations. Data: Median (IQR); *n* (%).

**Table 3:** Number of FI episodes in 3 weeks and score evolution. Each follow-up period has been compared with the baseline results.

	Baseline (*n*: 13)	1 m ^†^ (*n*: 13)	3 m (*n*: 13)	6 m (*n*: 13)	12 m (*n*: 13)	18 m (*n*: 11)	24 m (*n*: 10)
**St. Mark’s (0–24)**	8 (4)	4 (4) **	4 (4) *	5 (3) **	6 (8)	5 (6, 5)	6 (9, 5)
**Soiling**	7 (18)	1 (3) *	0 (0) *	0 (3) *	2 (4) *	2 (6) *	1 (7)
**Urgency**	0 (4)	0 (0)	0 (0)	0 (0)	0 (2)	0 (4, 5)	3 (11, 25)
**Gas FI ^§^**	13 (13)	4 (13)	6 (0) *	4 (12) *	4 (10) *	1 (18) *	3 (21)
**Liquid FI**	0 (0)	0 (0)	0 (0)	0 (0)	0 (0)	0 (0)	0 (1)
**VAS ^‡^** **(0–10)**	3 (2, 5)	7 (4) **	4 (4)	5 (3) *	7 (1, 5) *	7(6,5) **	6 (9, 5) *

Values: Median (IQR), ^†^ m: months, ^‡^ VAS: visual analogue scale, ^§^ FI: faecal incontinence, * *p* < 0.05, ** *p* < 0.001 (Wilcoxon test).

**Table 4:** Clinical evolution from baseline to the last follow-up.

	Baseline GI ^§ †^	Last F-Up ^¶^ GI ^†^	Baseline Soiling ^†^	Last F-Up Soiling ^†^	>70% GI Improvement YES/NO	>70% Soiling Improvement YES/NO	Baseline VAS ^††^	Last F-Up VAS	Would You Repeat the Treatment?
**Patient 1**	8	0	21	0	Yes	Yes	5	7	No
**Patient 2**	8	0	12	8	Yes	No	0	6	Yes
**Patient 3**	21	21	21	0	No	Yes	2	6	Yes
**Patient 4**	10	21	7	21	No	No	1	6	No
**Patient 5**	7	0	4	0	Yes	Yes	4	7	Yes
**Patient 6**	21	21	3	0	No	Yes	6	6	Yes
**Patient 7**	21	18	7	0	No	Yes	3	7	No
**Patient 8**	21	21	0	0	No	-	3	1	Yes
**Patient 9**	21	8	14	2	No	Yes	6	6	Yes
**Patient 10**	13	6	0	0	Yes	-	3	7	Yes
**Patient 11**	21	0	21	6	Yes	Yes	4	6	Yes
**Patient 12**	0	0	21	6	Yes	Yes	5	6	Yes
**Patient 13**	2	0	3	3	No	No	5	5	No

^§^ GI: gas incontinence. ^†^ Days with symptoms in a three-week continence diary. ^¶^ f-up: follow-up; ^††^ VAS: visual analogue scale.

**Table 5:** Pre- and post-Gatekeeper manometric results.

	Pre-Treatment	Post-Treatment	*p* *
Mean resting pressure (mmHg)	34 (19)	29.4 (6, 5)	0.2 (n.s)
Mean squeeze pressure (mmHg)	73 (55)	64 (41)	0.1 (n.s)
**Rectal Sensitivity**			
First sensation volume (mL)	15 (11, 25)	20 (22, 5)	0.02 **
Desire to defecate volume (mL)	50 (18, 75)	60 (27, 5)	0.1 (n.s)
Maximum Tolerated Volume (mL)	95 (13, 75)	80 (25)	0.9 (n.s)

Data Median (IQR); * Wilcoxon Test.** *p* < 0.05; n.s. not significant.

## Data Availability

The data presented in this study are available upon request to correspondence authors.

## References

[1] Maestre Y., Parés D., Vial M., Bohle B., Sala M., Grande L. (2010). Prevalence of fecal incontinence and its relationship with bowel habit in patients attended in primary care. Med. Clin..

[2] Sharma A., Yuan L., Marshall R.J., Merrie A.E., Bissett I.P. (2016). Systematic review of the prevalence of faecal incontinence. Br. J. Surg..

[3] Assmann S.L., Keszthelyi D., Kleijnen J., Anastasiou F., Bradshaw E., Brannigan A.E., Carrington E.V., Chiarioni G., Ebben L.D., Gladman M.A. (2022). Guideline for the diagnosis and treatment of Faecal Incontinence-A UEG/ESCP/ESNM/ESPCG collaboration. United Eur. Gastroenterol. J..

[4] Falletto E., Martellucci J., Rossitti P., Bondurri A., Zaffaroni G., Ascanelli S., Chimisso L., Lauretta A., Mirafiori M., Clementi I. (2023). Transanal irrigation in functional bowel disorders and LARS: Short-term results from an Italian national study. Tech. Coloproctol..

[5] Shafik A. (1995). Perianal injection of autologous fat for treatment of sphincteric incontinence. Dis. Colon Rectum.

[6] Kumar D., Benson M.J., Bland J.E. (1998). Glutaraldehyde cross-linked collagen in the treatment of faecal incontinence. Br. J. Surg..

[7] Vaizey C.J., Kamm M.A. (2005). Injectable bulking agents for treating faecal incontinence. Br. J. Surg..

[8] Tjandra J.J., Lim J.F., Hiscock R., Rajendra P. (2004). Injectable silicone biomaterial for fecal incontinence caused by internal anal sphincter dysfunction is effective. Dis. Colon Rectum.

[9] Siproudhis L., Morcet J., Lainé F. (2007). Elastomer implants in faecal incontinence: A blind, randomized placebo-controlled study. Aliment. Pharmacol. Ther..

[10] Maeda Y., Laurberg S., Norton C. (2013). Perianal injectable bulking agents as treatment for faecal incontinence in adults. Cochrane Database Syst. Rev..

[11] Davis K., Kumar D., Poloniecki J. (2003). Preliminary evaluation of an injectable anal sphincter bulking agent (Durasphere) in the management of faecal incontinence. Aliment. Pharmacol. Ther..

[12] Ganio E., Trompetto M., Realis Luc A., Clerico G. (2004). Initial clinical results using Coaptite for the treatment of fecal incontinence. Dis. Colon Rectum.

[13] Maeda Y., Vaizey C.J., Kamm M.A. (2008). Pilot study of two new injectable bulking agents for the treatment of faecal incontinence. Color. Dis..

[14] Luo C., Samaranayake C.B., Plank L.D., Bissett I.P. (2010). Systematic review on the efficacy and safety of injectable bulking agents for passive faecal incontinence. Color. Dis..

[15] Hussain Z.I., Lim M., Stojkovic S.G. (2011). Systematic review of perianal implants in the treatment of faecal incontinence. Br. J. Surg..

[16] Hong K.D., Kim J.S., Ji W.B., Um J.W. (2017). Midterm outcomes of injectable bulking agents for fecal incontinence: A systematic review and meta-analysis. Tech. Coloproctol..

[17] Ratto C., Parello A., Donisi L., Litta F., De Simone V., Spazzafumo L., Giordano P. (2011). Novel bulking agent for faecal incontinence. Br. J. Surg..

[18] Cuschieri S. (2019). The STROBE guidelines. Saudi J. Anaesth..

[19] Ratto C., Buntzen S., Aigner F., Altomare D.F., Heydari A., Donisi L., Lundby L., Parello A. (2016). Multicentre observational study of the Gatekeeper for faecal incontinence. Br. J. Surg..

[20] Vaizey C.J., Carapeti E., Cahill J.A., Kamm M.A. (1999). Prospective comparison of faecal incontinence grading systems. Gut.

[21] Jabbar S.A.A., Camilleri-Brennan J. (2022). An evaluation of the long-term effectiveness of Gatekeeper™ intersphincteric implants for passive faecal incontinence. Tech. Coloproctol..

[22] Trenti L., Biondo S., Noguerales F., Nomdedeu J., Coret A., Scherer R., Fraccalvieri D., Frago R., Kreisler E. (2017). Outcomes of Gatekeeper TM prosthesis implantation for the treatment of fecal incontinence: A multicenter observational study. Tech. Coloproctol..

[23] Altomare D.F., Giuratrabocchetta S., Knowles C.H., Muñoz Duyos A., Robert-Yap J., Matzel K.E., European SNS Outcome Study Group (2015). Long-term outcomes of sacral nerve stimulation for faecal incontinence. Br. J. Surg..

[24] Duelund-Jakobsen J., van Wunnik B., Buntzen S., Lundby L., Laurberg S., Baeten C. (2014). Baseline factors predictive of patient satisfaction with sacral neuromodulation for idiopathic fecal incontinence. Int. J. Color. Dis..

[25] Grossi U., De Simone V., Parello A., Litta F., Donisi L., Di Tanna G.L., Goglia M., Ratto C. (2019). Gatekeeper Improves Voluntary Contractility in Patients with Fecal Incontinence. Surg. Innov..

[26] de la Portilla F., Reyes-Díaz M.L., Maestre M.V., Jiménez-Rodríguez R.M., García-Cabrera A.M., Vázquez-Monchul J.M., Díaz-Pavón J.M., Padillo-Ruiz F.C. (2017). Ultrasonographic evidence of Gatekeeper™ prosthesis migration in patients treated for faecal incontinence: A case series. Int. J. Color. Dis..

[27] Brusciano L., Tolone S., Del Genio G., Grossi U., Schiattarella A., Piccolo F.P., Martellucci J., di Visconte M.S., Docimo L. (2020). Middle-term Outcomes of Gatekeeper Implantation for Fecal Incontinence. Dis. Colon Rectum.

[28] Grossi U., Brusciano L., Tolone S., Del Genio G., Di Tanna G.L., Gambardella C., Docimo L. (2020). Implantable Agents for Fecal Incontinence: An Age-Matched Retrospective Cohort Analysis of GateKeeper versus SphinKeeper. Surg. Innov..

[29] Baig M.K., Wexner S.D. (2000). Factors predictive of outcome after surgery for faecal incontinence. Br. J. Surg..

[30] Rockwood T.H., Church J.M., Fleshman J.W., Kane R.L., Mavrantonis C., Thorson A.G., Wexner S.D., Bliss D., Lowry A.C. (2000). Fecal Incontinence Quality of Life Scale: Quality of life instrument for patients with fecal incontinence. Dis. Colon Rectum.

[31] Ware J.E., Sherbourne C.D. (1992). The MOS 36-item short-form health survey (SF-36). I. Conceptual framework and item selection. Med. Care.

[32] Litta F., Marra A.A., Ortega Torrecilla N., Orefice R., Parello A., De Simone V., Campennì P., Goglia M., Ratto C. (2021). Implant of Self-Expandable Artificial Anal Sphincter in Patients with Fecal Incontinence Improves External Anal Sphincter Contractility. Dis. Colon Rectum.

[33] Dawoud C., Bender L., Widmann K.M., Harpain F., Riss S. (2021). Sphinkeeper Procedure for Treating Severe Faecal Incontinence—A Prospective Cohort Study. J. Clin. Med..

[34] Litta F., Parello A., De Simone V., Campennì P., Orefice R., Marra A.A., Goglia M., Moroni R., Ratto C. (2020). Efficacy of Sphinkeeper™ implant in treating faecal incontinence. Br. J. Surg..

